# Determination of the Number of Circulating Small Extracellular Vesicles in Pregnancy Using the Novel Marker CD9

**DOI:** 10.3390/ijms262210906

**Published:** 2025-11-10

**Authors:** Risa Narumi, Hirotada Suzuki, Manabu Ogoyama, Yasushi Saga, Shohei Tozawa, Syunya Noguchi, Akihide Ohkuchi, Toshihiro Takizawa, Hiroyuki Fujiwara, Hironori Takahashi

**Affiliations:** 1 Department of Obstetrics and Gynecology, Jichi Medical University, Tochigi 329-0498, Japan99019mo@jichi.ac.jp (M.O.); hironori@jichi.ac.jp (H.T.); 2 Department of Molecular Medicine and Anatomy, Nippon Medical School, Tokyo 113-8602, Japan; n-syunya@nms.ac.jp (S.N.); t-takizawa@nms.ac.jp (T.T.)

**Keywords:** biomarker, CD9, small extracellular vesicles, exosome, preeclampsia

## Abstract

Small extracellular vesicles (small EVs) play pivotal roles in intercellular communication and pregnancy maintenance, but their clinical significance in preeclampsia (PE) remains unclear. We obtained plasma samples from non-pregnant women, healthy pregnant women, and patients with early-onset (EoPE) and late-onset PE (LoPE). Small EVs were isolated using ultracentrifugation and validated using transmission electron microscopy and nanoparticle tracking analysis; in addition, Western blotting was performed to identify suitable surface markers for plasma-derived small EVs. In our analysis, we consistently detected cluster of differentiation 9 (CD9), whereas classical markers such as cluster of differentiation 63 (CD63) and tumor susceptibility gene 101 (TSG101) were absent. In a prospective, nested case–control study, we analyzed first-trimester samples by using a CD9-based ELISA for small-EV quantification. The number of small EVs did not significantly differ between non-pregnant and healthy pregnant women regardless of the gestational age. However, EVs were significantly elevated in both EoPE (3.5-fold) and LoPE (1.5-fold) compared with matched controls. First-trimester EV levels did not show differences between women who later developed PE and normal controls. These findings indicate that CD9 is a promising marker for plasma-derived small EVs and that an elevated number of small EVs is associated with established PE but has limited predictive value in early pregnancy. Further studies are required to elucidate the cellular origin and clinical implications of small EVs in PE.

## 1. Introduction

Small extracellular vesicles (small EVs) are 30–200 nm bioactive particles that play crucial roles in cell–cell communication by carrying proteins, lipids, and nucleic acids, including microRNAs [1,2,3]. During pregnancy, small EVs are secreted by trophoblasts, and flow into maternal circulation [4,5]. They play an essential role in communication between the fetus and the mother, contributing to key processes such as embryo implantation, modulation of the maternal immune response, and placental development [6]. Their secretion quantity is one of the main factors in small EV-related biology/pathology and changes based on the cell type and pathological conditions (e.g., cancer and autoimmune disease) [7,8,9,10]; patients with systemic lupus erythematosus (SLE) show a higher number of serum-derived small EVs than healthy controls [11]. In pregnancy, the quantity of serum/plasma-derived small EVs increases as pregnancy progresses [12,13,14]. Furthermore, the quantity of plasma-derived small EVs increases in patients with preeclampsia (PE) [15,16,17]. Preeclampsia is a chronic inflammatory endothelial disorder caused by factors released from the ischemic placenta during early pregnancy. Small EVs are thought to meditate this pathology by enhancing immune activation, inflammation, and endothelial injury [18]. The number of small EVs is usually measured with quantitative analyses (e.g., ELISA) using specific markers [19]. However, the identification of such specific markers remains a subject of debate. A recent study in a high-impact journal investigated EVs and their markers in several materials, including cell lines, tissue explants, serum, plasma, lymphatic fluid, bone marrow, and biliary liquid [20], and found that the positive rate of the markers differed by origin or cell. The most useful biomarker that physicians use is blood (serum and plasma), and CD63 has been the most widely used small-EV marker. However, according to the above-mentioned study [20], CD63 was negative in blood (serum and plasma)-derived small EVs. We aimed to conduct a re-evaluation of small EVs and their markers in maternal blood during pregnancy, and to this end, measured their quantity in maternal plasma by using a small-EV marker other than CD63. Furthermore, we examined the quantity of plasma-derived small EVs in patients with PE compared with normotensive controls.

## 2. Results

### 2.1. Patient Characteristics

Our study followed two designs: a case–control study and a prospective, nested case–control study. Firstly, in the case–control study (patient characteristics are shown in Table 1), we compared patients with early onset preeclampsia (EoPE) and normotensive controls. Between EoPE patients and normotensive controls in the second trimester, systolic blood pressure (median of 165 [interquartile range (IQR) 161–176] vs. 116 [108–123] mmHg, respectively; *p* < 0.001), diastolic blood pressure (100 [89.5–108.5] vs. 72 [62–79] mmHg, respectively; *p* < 0.001), and serum creatinine levels (0.70 [0.50–0.79] vs. 0.49 [0.39–0.52] mg/dL, respectively; *p* = 0.03) were significantly elevated in the former (Table 1); additionally, in EoPE patients, the gestational age at delivery was significantly earlier (33.6 [33.0–34.3] vs. 39.3 [38.1–40.6] weeks, respectively; *p* < 0.001), and birth weight was significantly lower (1750 [1292–1904] vs. 2994 [2842–3156] grams, respectively; *p* < 0.001) (Table 1). Between late-onset preeclampsia (LoPE) patients and normotensive controls in the third trimester, systolic blood pressure (154 [151–170] vs. 117 [106–126] mmHg, respectively; *p* < 0.001) and diastolic blood pressure (98 [90–102] vs. 71 [64–84] mmHg, respectively; *p* < 0.001) were significantly elevated in the former (Table 1); additionally, in LoPE patients, the gestational age at delivery was significantly earlier (38.0 [36.8–38.5] vs. 39.6 [39.1–40.8] weeks, respectively; *p* = 0.004), and birth weight was significantly lower (2813 [2299–2988] vs. 3156 [2839–3414] grams, respectively; *p* = 0.03) (Table 1). Secondly, in the prospective, nested case–control study (patient characteristics are shown in Table 2), we prospectively included singleton pregnant women in the first trimester, from whom we collected blood samples, and then categorized them into three groups based on the perinatal outcome (normal controls, EoPE, and LoPE). There were no significant differences in maternal age, BMI, or blood pressure among the three groups (Table 2), while the serum creatine level showed a significant increase in patients with LoPE compared with the normotensive controls (Table 2).

### 2.2. Characterization and Evaluation of Circulating Small EVs

**Figure 1:**
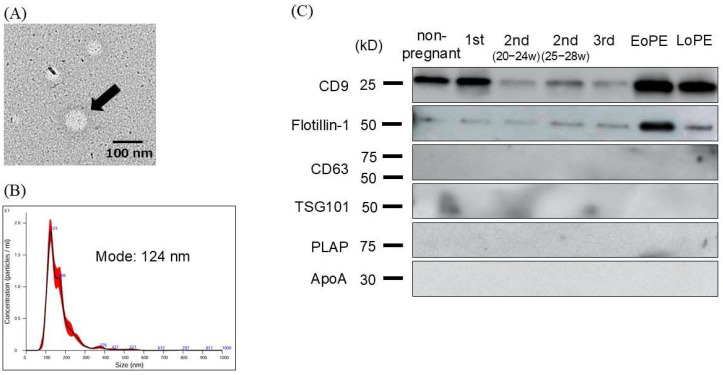
Validation of small extracellular vesicles (EVs) derived from plasma of non-pregnant and pregnant women. (**A**) EVs in transmission electron microscopy (TEM), which revealed that the EV diameter (arrow) was approximately 100 nm. (**B**) Nanoparticle tracking analysis (NTA) to identify small EVs, where we found that the mode size of the isolated small EVs was 124 nm. (**C**) Western blotting analysis of isolated small EVs, where multiple small-EV markers were examined.

We evaluated purified small EVs derived from maternal plasma by using several methods. We confirmed the presence of particles approximately 100 nm in diameter with transmission electron microscopy (Figure 1A), and nanoparticle tracking analysis (NTA) revealed that the majority of particles ranged from 110 to 140 nm in diameter (Figure 1B). Next, we explored small-EV markers in maternal plasma with Western blotting, which showed positive signals for CD9 and Flotillin-1, while CD63, TSG101, and placental alkaline phosphatase (PLAP), which are known as small-EV markers derived from other tissues, were negative. Apolipoprotein A1 (ApoA1), which is known as one of the negative markers of small EVs [21], was also negative (Figure 1C).

### 2.3. The Number of Small EVs in Non-Pregnant Women and Normotensive Controls

**Figure 2:**
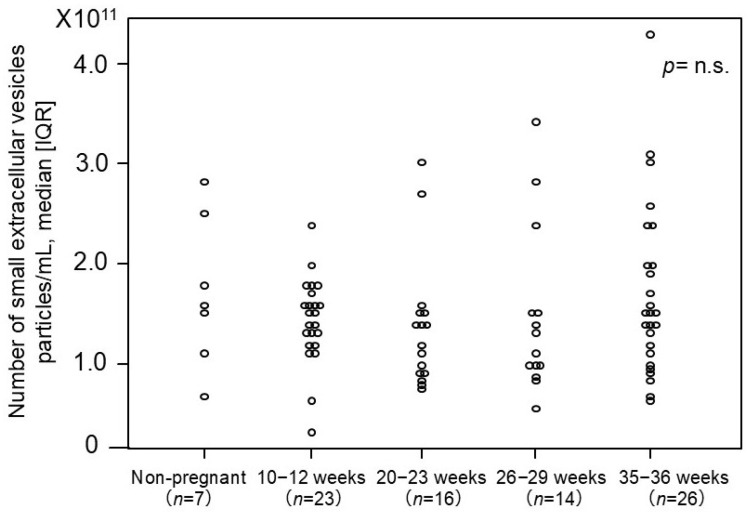
The number of plasma-derived small EVs from non-pregnant and pregnant women. The median [IQR] numbers (particles/mL) were as follows: 1.6 × 10^11^ [1.3 × 10^11^–2.2 × 10^11^] particles/mL in non-pregnant women, 1.5 × 10^11^ [1.3 × 10^11^–1.7 × 10^11^] in pregnant women at 10–12 weeks, 1.3 × 10^11^ [9.0 × 10^10^–1.5 × 10^11^] in pregnant women at 20–23 weeks, 1.2 × 10^11^ [1.0 × 10^11^–1.5 × 10^11^] in pregnant women at 26–29 weeks, and 1.5 × 10^11^ [1.1 × 10^10^–2.0 × 10^11^] in pregnant women at 35–36 weeks. CD9 was employed as the small-EV marker in this analysis. IQR: interquartile range. *p*-value = n.s. (no significant).

The number of small EVs was measured using an ELISA-based method targeting the surface marker CD9 based on the above-mentioned experiments (Figure 2). No significant differences in the number of small EVs were observed among the normotensive controls across varying gestational ages, nor between non-pregnant women and normotensive controls.

### 2.4. Comparison of Number of Small EVs Between Patients with PE and Normotensive Controls

**Figure 3:**
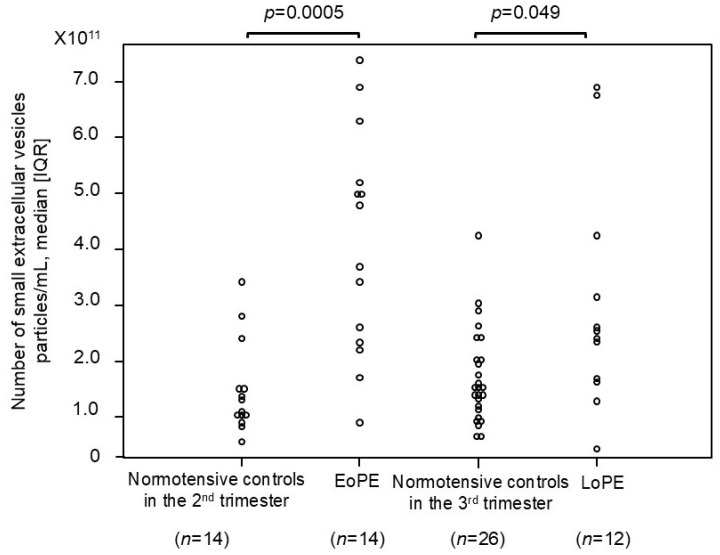
The number of small EVs in patients with preeclampsia and normotensive controls. The median [IQR] numbers (particles/mL) were as follows: 1.2 × 10^11^ [1.0 × 10^11^–1.5 × 10^11^] in normotensive controls in the 2nd trimester, 4.3 × 10^11^ [2.4 × 10^11^–5.2 × 10^11^] in patients with early onset preeclampsia, 1.5 × 10^11^ [1.1 × 10^11^–2.0 × 10^11^] in normotensive controls in the 3rd trimester, and 2.3 × 10^11^ [1.6 × 10^11^–3.4 × 10^11^] in patients with late-onset preeclampsia. CD9 was employed as the small-EV marker in this analysis. IQR: interquartile range; EoPE: early onset preeclampsia; LoPE: late-onset preeclampsia.

The number of small EVs was compared between patients with EoPE and normotensive controls (Figure 3), and we found that it was significantly higher, approximately 3.5-fold, in the former than in the normotensive controls during the second trimester (4.3 × 10^11^ [2.4 × 10^11^–5.2 × 10^11^] vs. 1.2 × 10^11^ [1.0 × 10^11^–1.5 × 10^11^] particles/mL, respectively; *p* = 0.0005). Similarly, the number of small EVs was significantly higher, approximately 1.5-fold, in patients with LoPE than in the normotensive controls during the third trimester (2.3 × 10^11^ [1.6 × 10^11^–3.4 × 10^11^] vs. 1.5 × 10^11^ [1.1 × 10^11^–2.0 × 10^11^] particles/mL, respectively; *p* = 0.049).

### 2.5. Comparison of Number of Small EVs in First Trimester Between Women Who Later Developed PE and Normotensive Controls in Prospective, Nested Case–Control Study

**Figure 4:**
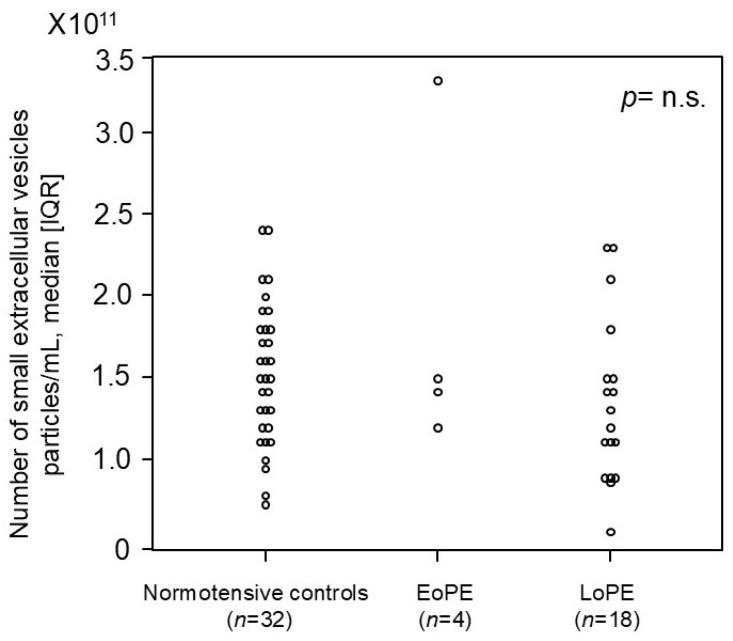
The number of small EVs in the first trimester in pregnant women who later developed preeclampsia and normotensive controls in the prospective cohort study. The median [IQR] numbers (particles/mL) were as follows: 1.5 × 10^11^ [1.2 × 10^11^–1.8 × 10^11^] in normotensive controls, 1.5 × 10^11^ [1.4 × 10^11^–2.0 × 10^11^] in patients who later developed early onset preeclampsia, and 1.3 × 10^11^ [9.4 × 10^10^–1.5 × 10^11^] in patients who later developed late-onset preeclampsia. CD9 was employed as the small-EV marker in this analysis. IQR: interquartile range; EoPE: early onset preeclampsia; LoPE: late-onset preeclampsia. *p*-value = n.s. (no significant).

We investigated whether the number of small EVs in the first trimester could serve as a potential biomarker for PE (Figure 4). We prospectively collected more than 1400 blood samples from pregnant women in the first trimester, among whom 4 cases with EoPE and 18 cases with LoPE later occurred, and measured the number of small EVs in each group. No significant difference was observed in the number of small EVs between women who later developed PE (early and late onset) and normotensive controls.

## 3. Discussion

We obtained several novel findings. First, the number of small EVs in human plasma was significantly increased in both early and late-onset PE compared with the healthy controls. Second, CD9 may be a more useful small-EV marker in maternal plasma than CD63, which is currently the most widely used marker. Furthermore, there were no significant changes in the number of small EVs throughout the stages of pregnancy, which is inconsistent with previous studies [12,13,14].

Regardless of onset time, the number of small EVs increased in patients with PE compared with gestational-age-matched normotensive controls. Several studies reported that the number of small EVs in plasma increased in the presence of not only PE but also several malignancies [22,23,24]. Matsumoto et al. [9] demonstrated that the number of serum EVs significantly increased in patients with esophageal cancer compared with healthy controls. Similar findings have also been reported in patients with lung [25,26] and prostate [27,28] cancer, as well as in those with autoimmune disease [11]. Regarding isolating small EVs, some of the above-mentioned studies did not employ ultracentrifugation but instead used polymer precipitation [9,11]. Although small EVs are easily collected with this method, the rate of contamination from other particles is high [29]. In our study, the isolated EVs were validated using both electron microscopy and nanoparticle tracking analysis (NTA); thus, we considered that we collected small EVs with good purity. Several mechanisms suggest that the number of small EVs increases in PE [30]. In PE, impaired placental perfusion leads to hypoxia, which induces stress in syncytiotrophoblasts and cytotrophoblasts, resulting in increased release of small EVs [31,32], a more apparent phenomenon in early than late-onset PE. Clinical symptoms associated with placental insufficiency (e.g., fetal growth restriction and oligohydramnios) can readily manifest in early onset PE, which indicates that syncytiotrophoblasts can have deleterious effects on maternal cells. When plasma-derived small EVs in patients with PE were applied to human aortic endothelial cells (HAECs), their migration was significantly reduced [16], and treatment with these EVs downregulated endothelial nitric oxide synthase (eNOS). Moreover, serum-derived EVs from patients with PE contain increased levels of *miR-155*, which suppresses eNOS expression in human umbilical vein endothelial cells (HUVECs) [33]. Plasma-derived small EVs from patients with PE are enriched in sFlt-1 and sEng, leading to HUVEC dysfunction [34].

CD9 may be a promising marker for small EVs in plasma. We performed Western blotting for multiple small-EV markers. Interestingly, classical small-EV markers including CD63 and TSG101 were undetectable in plasma-derived small EVs, whereas CD9 was reliably detected. Our findings are supported by a large-scale study [20]; the authors, however, did not investigate maternal samples but isolated small EVs from the plasma of 120 people and evaluated the samples for the expression of 11 classical small-EV markers by using proteomic techniques. Surprisingly, no samples (0%) were positive for CD63, heat shock protein 70 (Hsp70), or TSG101, among which CD63 is one of the most widely used small-EV markers [11,12,15] detectable in EVs derived from cell lines or tissue explants. While the above were not detected in blood-derived EVs (serum or plasma), CD9 showed the highest positive rate, as high as 62%, followed by heat shock protein family A member 8 (HSPA8) (58%). These results indicate that CD9 is the most reliable marker of plasma-derived small EVs. The above-mentioned comprehensive study also proposed fibronectin1 (FN1) as a promising novel marker for blood-derived small EVs, showing a 100% positive rate, among other novel markers. Future analyses of blood-derived small EVs should consider focusing on CD9 as a primary marker, along with novel markers for accuracy and comprehensive characterization.

Unlike previous reports showing that the levels of small EVs increase as pregnancy progresses [12,13,14], our study found no significant changes in the number of EVs. There are several possible explanations for this difference. First, previous studies used small-EV markers (e.g., PLAP and CD63) and isolation methods (e.g., commercial kits and density gradients) that differed from those in our study. Second, small EVs are abundantly secreted by cells under hypoxic or inflammatory conditions [31,35]; therefore, the number of small EVs is elevated in PE, which is associated with early placental ischemia and chronic inflammation. Contrarily, the phenomenon was not observed in normal pregnancies, explaining why the number of EVs was not elevated in our study. Third, as pregnancy progresses, circulating blood volume significantly increases; thus, even if placenta-derived small EVs increase in number, the quantity of small EVs per unit volume may remain unchanged.

PE prediction using small EVs remains challenging. We investigated the number of plasma-derived small EVs as a predictive biomarker for the development of PE in the first trimester and observed a declining tendency in patients with LoPE, although the sample number was small. The sFlt-1/PlGF ratio is a useful biomarker for predicting PE, but its use is limited to women in the second–third trimesters [36]. In contrast, small EVs may hold promise as predictive markers from early pregnancy. Identifying patients at high risk of PE in the first trimester would be of marked clinical value, because low-dose aspirin can significantly reduce such risk. Currently, the isolation of small EVs remains time-consuming and limited in throughput, which has constrained its clinical applicability. However, future technological advances are expected to facilitate the clinical implementation of small-EV-based approaches.

This study has two strengths. First, we carefully performed EV isolation and successfully obtained high-quality samples. As commercial kits offer convenience but may introduce contaminants, we extensively validated different isolation methods; for the first time, we demonstrated that CD9 is particularly useful, which is the main strength of our study. Second, samples from pregnant women were collected as part of a prospective cohort study. The incidence of early onset PE is approximately 0.5%, which means that about 200 participants were required to obtain a single case. Consequently, this process required considerable effort, and the data could not be easily replicated or followed-up by other groups.

There were several limitations in this study. First, although CD9 was employed as a marker for small EVs, there is no evidence that CD9 is universally expressed in plasma-derived small EVs; thus, as above-mentioned, a multi-marker approach may be optimal. Second, we focused on the number of small EVs in blood. As a result, we did not analyze the EV contents and were thus unable to explore their biological or pathological effects. Third, although contamination by non-vesicular extracellular particles (NVEPs) should be carefully assessed [29], this issue was not evaluated in the present study. Finally, this was a single-center study with a limited number of cases.

In summary, the number of small EVs did not increase throughout pregnancy but significantly increased in patients with both early and late-onset PE. According to our findings, CD9 may be one of the most reliable markers of small EVs in human plasma. Small EVs may play important roles in pregnancy maintenance and pregnancy-related complications, including PE. Further investigations are needed.

## 4. Materials and Methods

### 4.1. Study Participants and Sample Collection

This study included singleton pregnant women whose pregnancy course was followed at our institution between July 2016 and August 2023. Plasma samples were collected from 7 non-pregnant women, 79 normotensive controls (10–12 weeks: 23 cases; 20–23 weeks: 16 cases; 26–29 weeks: 14 cases; 35–36 weeks: 26 cases), and 26 patients with PE (early onset: 14 cases; late-onset: 12 cases) to compare the number of small EVs. Furthermore, plasma samples from women who later developed PE (early onset: 4 cases; late-onset: 18 cases) and those who experienced an uncomplicated pregnancy (32 cases) in our cohort were used to investigate the difference in the number of small EVs. PE was defined as new-onset hypertension (systolic blood pressure ≥ 140 mmHg or diastolic blood pressure ≥ 90 mmHg) after 20 weeks of gestation, accompanied by proteinuria (≥300 mg/day), fetal growth restriction, or organ dysfunction (e.g., low platelet and liver dysfunction); these conditions return to normal within 12 weeks postpartum. Early onset PE was defined as onset before 34 weeks of gestation and late-onset PE as onset at or after 34 weeks of gestation [37]. Blood samples were collected in EDTA tubes and centrifuged at 2700 rpm for 10 min at 4 °C (model 5500, KUBOTA, Tokyo, Japan). The plasma was stored at −80 °C. All participants provided written informed consent approved by the institutional ethics committee.

### 4.2. Purification of Small EVs

Small EVs were purified from plasma using a sequential ultracentrifugation method, as described below. Briefly, 2 mL of plasma was diluted with an equal volume of phosphate-buffered saline (PBS) and centrifuged at 2000× *g* for 30 min at 4 °C. The supernatant was transferred to a new tube and centrifuged at 12,000× *g* for 45 min at 4 °C. The resulting supernatant was transferred to an ultracentrifuge tube (Cat. No. 32143650, Beckman Coulter, CA, USA) and ultracentrifuged at 110,000× *g* for 2 h at 4 °C (Optima L-90 Ultracentrifuge, Type SW41Ti swinging-bucket rotor, Beckman Coulter, CA, USA). The pellet was resuspended in 1 mL of PBS, passed through a 0.2 μm filter (Cat. No. S7597, Sartorius AG, Göttingen, Germany), transferred to a new ultracentrifuge tube, and again ultracentrifuged at 110,000× *g* for 70 min at 4 °C. The resulting pellet was resuspended in 1 mL of PBS and subjected to a final ultracentrifugation at 110,000× *g* for 70 min at 4 °C. The final pellet was resuspended in 150 μL of PBS and stored at −80 °C. The protein concentration of the purified small EVs was measured using the Qubit Protein Assay Kit (Cat. No. Q33212, Thermo Fisher Scientific, Waltham, MA, USA).

### 4.3. Western Blotting

The small EVs were solubilized in an equal volume of M-PER Mammalian Protein Extraction Reagent (Cat. No. 78501, Thermo Fisher Scientific, Waltham, MA, USA) and diluted with Laemmli Sample Buffer (4X) (Cat. No. 1610747, BIO-RAD, Hercules, CA, USA) and PBS to obtain a final protein concentration of 30 μg/lane. The samples were incubated at 95 °C for 5 min. The small-EV lysates were subjected to electrophoresis using Mini-PROTEAN TGX gels (Cat. No. 4561094, BIO-RAD, Hercules, CA, USA), followed by transfer onto PVDF membranes (Cat. No. 1704156, BIO-RAD, Hercules, CA, USA), which were blocked with 5% skim milk for 1 h at room temperature. The primary antibodies anti-CD9 (Cat. No. SHI-EXO-M01, COSMO BIO COMPANY, Tokyo, Japan), anti-Flotillin-1 (Cat. No. ab133497, Abcam, Cambridge, UK), anti-CD63 (Cat. No. ab68418, Abcam, Cambridge, UK), anti-TSG101 (Cat. No. ab125011, Abcam, Cambridge, UK), anti-PLAP (Cat. No. ab133602, Abcam, Cambridge, UK), and anti-ApoA1 (Cat. No. 14427-1-AP, Proteintech Group, Rosemont, IL, USA) were incubated overnight at 4 °C. Following washing with Tris-buffered saline containing Tween 20 (TBST), the membranes were incubated with horseradish peroxidase (HRP)-conjugated secondary antibodies—goat anti-rabbit IgG (Cat. No. SA00001-2, Proteintech Group, Rosemont, IL, USA) or goat anti-mouse IgG (Cat. No. SA00001-1, Proteintech Group, Rosemont, IL, USA)—for 1 h at room temperature. After washing the membranes with TBST, we added Western ECL Substrate (Cat. No. 1705062, BIO-RAD, Hercules, CA, USA), and signals were detected using the Amersham Imager 680 (Cytiva, Tokyo, Japan). All experiments were performed at least twice for each antibody.

### 4.4. Nanoparticle Tracking Analysis (NTA)

The particle size of the small EVs was measured using the Nanosight LM10 system (NTA software version: NTA 3.2 Dev Build 3.2.16). The small EVs were diluted with PBS and analyzed under the following settings: Camera Type: sCMOS; Camera Level: 14–15; Capture Time: 60 s; Number of Repeats: 5; Detection Threshold: 5.

### 4.5. Transmission Electron Microscopy (TEM)

A collodion-coated grid (Cat. No. 651, NISSHIN EM, Tokyo, Japan) was loaded with the small-EV solution, followed by negative staining using an equal volume of uranyl acetate solution (Cat. No. 336, NISSHIN EM, Tokyo, Japan). The size and morphology of the small EVs were observed using a transmission electron microscope (H-7600, Hitachi High-Tech, Tokyo, Japan).

### 4.6. Measurement of Small-EV Numbers

Small-EV numbers were measured using CD9, which was identified as the most potent and reliable surface marker for plasma-derived small EVs (as described in the Results section). The assay was performed according to the manufacturer’s instructions using an Enzyme-Linked Immunosorbent Assay (EXEL-ULTRA-CD9-1, System Biosciences, Palo Alto, CA, USA). Briefly, the plasma-derived small EVs and standard EVs (particle numbers determined with nanoparticle tracking analysis) were applied to the supplied 96-well plate and incubated for 1 h at 37 °C. After washing the plate with Wash Buffer, the CD9 primary antibody was added and incubated for 1 h at room temperature. The plate was washed again, followed by incubation with the secondary antibody for 1 h at room temperature. After a final wash with Wash Buffer, Super Sensitive TMB ELISA substrate was added and incubated for 15 min at room temperature. The reaction was stopped with Stop Buffer, and absorbance was measured at 450 nm.

### 4.7. Statistical Analysis

All data were analyzed using the statistical software tool EZR (Y. Kanda, Saitama Medical Center, Jichi Medical University, Saitama, Japan), which is a graphical user interface for R (version 3.0.2). Categorical variables were compared using Fisher’s exact test, while for continuous variables, the Mann–Whitney U test was applied for comparisons between two groups, and the Kruskal–Wallis test (with Steel–Dwass post hoc analysis) was used for comparisons among multiple groups. A *p*-value < 0.05 was considered significant.

## Figures and Tables

**Table 1:** Characteristics of patients with preeclampsia and normotensive controls.

	Normotensive Controls 2nd Trimester (*n* = 14)	EoPE (*n* = 14)	*p*-Value	Normotensive Controls 3rd Trimester (*n*=26)	LoPE (*n* = 12)	*p*-Value
Maternal age (years)	32.5 [30.3–34.0]	36.0 [31.5–39.5]	0.16	34.0 [31.5–38.5]	35.0 [32.5–36.8]	0.98
BMI (kg/m^2^)	20.4 [19.0–23.2]	22.6 [20.2–23.7]	0.19	23.2 [21.4–27.2]	24.5 [20.8–26.7]	0.65
Gestational age at blood collection (weeks)	26.9 [26.7–27.3]	31.4 [28.6–33.3]	0.003	36.9 [36.5–37.1]	36.6 [35.7–38.5]	0.94
Systolic blood pressure (mmHg)	116 [108–123]	165 [161–176]	<0.001	117 [106–126]	154 [151–170]	<0.001
Diastolic blood pressure (mmHg)	72 [62–79]	100 [89.5–108.5]	<0.001	71 [64–84]	98 [90–102]	<0.001
Proteinuria (g/day)	–	3.0 [1.6–5.8]	NA	–	1.2 [0.9–1.6]	NA
Serum creatinine (mg/dL)	0.49 [0.39–0.52]	0.70 [0.50–0.79]	0.03	0.55 [0.45–0.67]	0.61 [0.55–0.68]	0.31
Gestational age at onset (weeks)	–	31.7 [28.7–33.0]	NA	–	36.1 [35.1–37.5]	NA
Gestational age at delivery (weeks)	39.3 [38.1–40.6]	33.6 [33.0–34.3]	<0.001	39.6 [39.1–40.8]	38.0 [36.8–38.5]	0.004
Birth weight (g)	2994 [2842–3156]	1750 [1292–1904]	<0.001	3156 [2839–3414]	2813 [2299–2988]	0.03

Medians [IQRs]. EoPE: early onset preeclampsia; LoPE: late-onset preeclampsia; BMI: body mass index; NA: not available.

**Table 2:** Characteristics of patients in prospective, nested case–control study.

	Normotensive Controls (*n* = 32)	EoPE (*n* = 4)	LoPE (*n* = 18)	*p*-Value
Maternal age (years)	35.5 [30.8–39.0]	38.5 [36.8–39.2]	35.5 [32.3–37.0]	0.45
BMI (kg/m^2^)	21.7 [19.6–23.9]	21.5 [20.7–24.2]	22.2 [19.7–25.3]	0.9
Gestational age at blood collection (weeks)	10.7 [10.3–11.4]	11.4 [10.6–12.1]	11.4 [10.7–12.0]	0.27
Systolic blood pressure (mmHg)	116 [107–125]	118 [113–122]	116 [107–125]	0.98
Diastolic blood pressure (mmHg)	66 [57–73]	81 [70–84]	70 [63–76]	0.15
Proteinuria (g/day)	–	2.7 [1.8–4.5]	0.7 [0.4–2.1]	0.15
Serum creatinine (mg/dL)	0.50 [0.45–0.55]	0.75 [0.65–0.82]	0.63 [0.60–0.69]	<0.001 ^a^
Gestational age at onset (weeks)	–	30.2 [29.5–31.2]	37.5 [36.5–38.9]	0.002
Gestational age at delivery (weeks)	39.0 [38.0–40.0]	31.5 [31.0–32.6]	38.7 [36.8–40.0]	0.002 ^b,c^
Birth weight (g)	3091 [2891–3290]	1280 [1118–1482]	2802 [2620–2920]	<0.001 ^a,b,c^

Medians [IQRs]. EoPE: early onset preeclampsia; LoPE: late-onset preeclampsia; BMI: body mass index; ^a^: normotensive controls vs. LoPE; ^b^: normotensive controls vs. EoPE; ^c^: EoPE vs. LoPE.

## Data Availability

The original contributions presented in this study are included in the article. Further inquiries can be directed to the corresponding author.

## Abbreviations

The following abbreviations are used in this manuscript:

EV	Extracellular vesicle
EoPE	Early onset preeclampsia
LoPE	Late-onset preeclampsia
NTA	Nanoparticle tracking analysis
HAEC	Human aortic endothelial cell
HUVEC	Human umbilical vein endothelial cell
NIPT	Non-invasive prenatal testing
TEM	Transmission electron microscopy
